# Synthesis of Amphiphilic Polyacrylates as Peelable Coatings for Optical Surface Cleaning

**DOI:** 10.3390/ma17194813

**Published:** 2024-09-30

**Authors:** Daofeng Zhu, Hao Huang, Anqi Liang, Yanling Yang, Baohan He, Abbas Ahmed, Xiaoyan Li, Fuchuan Ding, Luyi Sun

**Affiliations:** 1 College of Chemistry and Materials Science & Fujian Key Laboratory of Polymer Science, Fujian Normal University, Fuzhou 350007, China; qsz20211446@student.fjnu.edu.cn (D.Z.); qsx20210995@student.fjnu.edu.cn (H.H.); qsx20220946@student.fjnu.edu.cn (A.L.); 107062020010@student.fjnu.edu.cn (Y.Y.); 107062020024@student.fjnu.edu.cn (B.H.); 2 Polymer Program, Institute of Materials Science and Department of Chemical & Biomolecular Engineering, University of Connecticut, Storrs, CT 06269, USA; abbas.ahmed@uconn.edu

**Keywords:** optical surface cleaning, amphiphilic polymer, peelable coating

## Abstract

Optical instruments require extremely high precision, and even minor surface contamination can severely impact their performance. Peelable coatings offer an effective and non-damaging method for removing contaminants from optical surfaces. In this study, an amphiphilic polyacrylate copolymer (PMLEA) was synthesized via solution radical copolymerization using the lipophilic monomer lauryl acrylate (LA) and hydrophilic monomers ER-10, methyl methacrylate (MMA), and butyl acrylate (BA). The structure and molecular weight of the copolymer were characterized using Fourier transform infrared spectroscopy (FTIR), nuclear magnetic resonance (NMR), and gel permeation chromatography (GPC). The hydrophilic–lipophilic balance, surface tension, and wettability of the copolymer were analyzed through water titration, the platinum plate method, and liquid contact angle tests. The cleaning performance of the copolymer coating on quartz glass surface contaminants was evaluated using optical microscopy and Ultraviolet-Visible Near-Infrared (UV-Vis-NIR) spectroscopy. The study examined the effect of varying the ratio of LA to ER-10 on the hydrophilicity, lipophilicity, cleaning efficiency, and mechanical properties of the copolymer coating. The results showed that when the mass ratio of LA to ER-10 was 1:2, the synthesized copolymer exhibited optimal performance in removing dust, grease, and fingerprints from quartz glass surfaces. The coating had a tensile strength of 2.57 MPa, an elongation at break of 183%, and a peeling force of 2.07 N m^−1^.

## 1. Introduction

The cleanliness of optical components’ surfaces is paramount. Contaminants can potentially absorb or scatter light, leading to the significant degradation of optical performance [1], including a decrease in transmittance [2], a reduction in laser-induced damage threshold [3,4], and serving as the initiators of laser-induced damage [5,6]. Dust, grease, and fingerprints represent the primary sources of contamination for optical devices [7,8]. Currently, dry-cleaning and wet-cleaning systems stand as the predominant methods for cleaning optical components [9,10]. Dry-cleaning techniques encompass methods such as dry ice blasting [11,12,13], mechanical wiping [14], and laser cleaning [8,15,16], while wet-cleaning includes steam cleaning [17] and solvent immersion cleaning [18]. However, dry-cleaning necessitates wiping the surface of the object, which poses a risk of scratching if there are particles of contaminants present. Conversely, after wet-cleaning, a residual smear forms a surface layer that can adversely affect the focus and contrast of optical elements [19,20]. In contrast, peelable coating cleaning technology offers several advantages, including high efficiency without secondary pollution, the avoidance of surface damage, and environmental friendliness [21].

Peelable coating serves as a temporary protective layer that can be easily removed from the substrate surface either at the end of its lifespan or after fulfilling its intended purpose [22]. During the removal process, it is crucial that the cohesive force within the strippable film exceeds the adhesive forces between the film and the substrate. This ensures that the film can be peeled off completely, leaving behind no residue [23,24]. Peeling off the functional coating and damaging the device’s surface would occur if the peeling force of the cleaning coating was greater than the functional coating’s adhesive force on the surface of the optical element [25].

Polymers commonly used in strippable coatings include vinyl polymers [26,27], polyurethanes [28,29], acrylics [30,31], and cellulose and its derivatives [32,33]. Choosing the right film-forming polymer and solvent for peelable coatings on optical surfaces is critical. Several conditions should be met by peelable cleaning coatings on optical surfaces [34]: rapid solvent-drying and short polymer-curing time, adaptability to different substrates, sufficient mechanical properties, the ease of application, quick and complete stripping, and protection of object surfaces while meeting cleaning requirements.

The effectiveness of cleaning depends on several factors, including temperature, hydrodynamic force, formulation, and cleaning time [35]. Contaminants on optical surfaces are often either hydrophilic or lipophilic. The amphiphilic nature of polymers facilitates the adhesion and encapsulation of contaminants, which increases the cleaning effectiveness [36,37].

Acrylate-based coatings are preferred for their durability, hardness, and scratch resistance, while the incorporation of copolymer monomers enhances functional versatility and adjustability compared to alternative coatings [38,39].

In this study, amphiphilic polyacrylates intended as film-forming materials were synthesized via solution polymerization. The primary monomers, methyl methacrylate and butyl acrylate, were combined with lauryl acrylate and ER-10 as functional monomers. Methyl methacrylate contributes strength, hardness, and adhesion to the coating, while butyl acrylate imparts flexibility, resulting in a balance of softness and hardness that yields excellent mechanical performance. Lauryl acrylate’s lengthy carbon chain endues the coating with lipophilicity and low adhesive force, which ensure that the cleaning coating can be easily removed without damaging the functional coating of the optical device, while the ER-10 surfactant adds hydroxyl groups to the resultant polymer to increase its degree of hydrophilicity. This coating improves the wetting, adhesion, and encapsulation of both aqueous and oily contaminants by enhancing the amphiphilicity of the copolymer. This allows it to effectively penetrate the interface between the contaminants and the material surface during cleaning, lifting the contaminants and incorporating them into the coating. When the coating is peeled away, the contaminants are removed along with it. The successful synthesis of the product was confirmed through the characterizations using Fourier transform infrared spectroscopy (FTIR), nuclear magnetic resonance (NMR), and gel permeation chromatography (GPC) techniques. The resulting coating exhibits a tensile strength of 3.47 MPa and a peel strength of less than 3.20 N m^−1^. These polyacrylate coatings demonstrate excellent performance in removing contaminants from quartz glass surfaces, leaving no visible residue under an optical microscope after cleaning. Furthermore, the transmittance of the cleaned quartz is restored to a level close to that of the original clean quartz.

## 2. Experiment

### 2.1. Materials

Methyl methacrylate (MMA), butyl acrylate (BA), 2,2′-azobis(2-methylpropionitrile) (AIBN), lauryl acrylate (LA), petroleum ether, propylene glycol methyl ether acetate (PMA), Span 80, Tween 20, ethanol, 1,4-dioxane, toluene, 1,3-dioxolane, and butanone were sourced from Sinopharm Chemical Reagent Co., Ltd. (Shanghai, China). The above reagents are analytically pure except for Span 80 and Tween 20, which are chemically pure. The SiO_2_ particles (AEROSIL^®^ R972) were from Evonik Industries AG (Essen, Germany). The silicone grease was from Sinopec Lubricants Co., Ltd. (Beijing, China). The ER-10 emulsifier was procured from Nippon Adeka Co., Ltd. (Tokyo, Japan). The specific structure of ER-10 is shown in Figure S2 (Support Information), where R is an alkyl group.

### 2.2. Preparation of Polyacrylates

**Figure 1:**
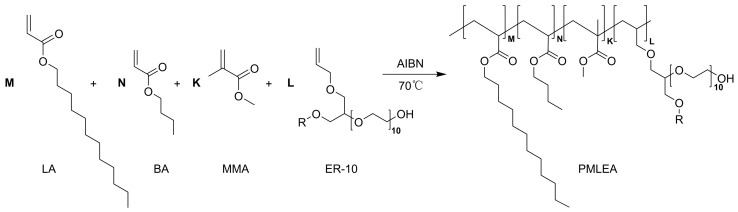
Synthesis reaction of PMLEA.

The polyacrylates were synthesized via solution polymerization using MMA, BA, LA, and ER-10 as monomers. The synthetic formulations are detailed in Table 1. The synthesis of polyacrylates is outlined in Figure 1, while the preparation details are provided in Supplementary Figure S1. The monomers, along with 38.40 g of PMA as the solvent, were combined in a 100.0 mL flask. The reaction was conducted in a water bath at 70 °C for 5 h with AIBN as the initiator. After the reaction, the polyacrylate sample was purified by first mixing the reacted solution into a 50% (*v*/*v*) water–ethanol solution to precipitate white flocculent. Subsequently, the product underwent washing with petroleum ether three times, followed by soaking in methanol for 24 h to obtain the purified polyacrylate sample. Finally, the polyacrylate after vacuum drying at 50 °C was dissolved in a 1:1 mass ratio of 1,3-dioxolane/butanone mixture to achieve a solution of 10 wt%.

### 2.3. Characterization and Performance Evaluation

#### 2.3.1. FTIR and NMR Characterization

The Fourier transform infrared (FTIR) spectra of approximately 5.0 μm thick polyacrylate films coated on single-crystal silicon wafers were collected using a Nicolet iS50 spectrophotometer (Thermo Fisher Scientific, Waltham, MA, USA) with a wavelength scanning range of 4000–400 cm^−1^. The NMR spectra of the polyacrylates were obtained using a Bruker 400 MHz spectrometer (Billerica, MA, USA), employing CDCl_3_ as the solvent.

#### 2.3.2. Gel Permeation Chromatography (GPC)

The gel permeation chromatography of the copolymer samples was performed using an Agilent 1260 (Santa Clara, CA, USA) liquid chromatograph equipped with a refractive index (RI) detector. The column setup included Agilent PLgel 5 μm Mixed-c, 10 μm Mixed-b, and PLgel 5 μm Guard in series. THF was used as the mobile phase with a flow rate set at 1 mL min^−1^.

#### 2.3.3. Differential Scanning Calorimetry (DSC)

The glass transition temperatures of the polyacrylates were determined using DSC (METTLER DSC3, Greifensee, Switzerland) in a temperature range of −20 to 200 °C at a rate of 10 °C/min under a N_2_ atmosphere.

#### 2.3.4. Hydrophilic–Lipophilic Balance (HLB) of the Polyacrylates

HLB, initially proposed by Griffin [40,41], represents an equilibrium relationship between the hydrophilic and lipophilic groups in surfactants concerning their size and strength. In this study, the HLBs of the polyacrylates were determined using the aqueous titration method developed by Greenwald [42]. The specific test procedures are as follows: A total of 1.00 g of a mixture of Span 80 and Tween 20 at various mass ratios was charged into a 100 mL conical flask, and then 30.0 mL of a mixture of 1,4-dioxane and toluene with a volume ratio of 96:4 was added to the conical flask. The solution was titrated with deionized water by shaking the conical flask until the solution became cloudy at 30 °C. The volume of titrating water was recorded as W_1_. Then, the relationship between log(W_1_) and HLB was plotted using a polynomial equation. The water volume (W_2_) required for the polymer solution of the 1,4-dioxane and toluene mixture was determined in the same way. Finally, the HLB of each polyacrylate was calculated by substituting W_2_ into the fitting Equation (1).

(1) $$Y=A_{1}*x^{3}+A_{2}*x^{2}+A_{3}*x+Intercept$$

#### 2.3.5. Surface Tension of Polymer Solutions

The surface tension of each polyacrylate solution was measured as the stable test values recorded by an automatic surface tension meter (K20, Kruss, Hamburg, Germany) using a platinum plate (24.0 mm × 10.0 mm × 0.1 mm) in contact with the surface of 10 wt% polyacrylate solution in a 1:1 mass ratio of 1,3-dioxolane/butanone mixture.

#### 2.3.6. Polyacrylate Wettability and Amphiphilicity

The wettability of each polyacrylate solution was characterized by recording the contact angle after 3 s for a drop of the polymer solution placed on the quartz glass surface using a syringe with a contact angle tester (DSA25E, Kruss, GER).

Additionally, the amphiphilicity of each polyacrylate was assessed by measuring the contact angles of 1.0 μL drops of deionized water and diiodomethane on the surface of the polyacrylate coating using a contact angle tester (DSA25E, Kruss, GER).

#### 2.3.7. Tensile Properties of the Polyacrylate Coatings

The tensile strength of the polyacrylates, which were sized at 70.0 mm × 25.0 mm × 0.1 mm, was tested using a universal testing machine (LR5K Plus, LLOYD, Bognor Regis, UK) equipped with a 100 N load cell. The test was conducted at a rate of 50.0 mm/min.

#### 2.3.8. The 180° Peel Test of Polyacrylate Coatings

The 180° peel test of the polyacrylate coatings involved peeling off coatings sized at 50.0 mm × 25.0 mm × 0.05 mm at a 180° angle, with a speed of 10.0 mm/min, using a universal testing machine (LR5K Plus, LLOYD, UK) equipped with a 100 N load cell.

#### 2.3.9. Coating Cleaning Effect Evaluation

In this study, three types of contamination: SiO_2_ particles, silicone grease, and fingerprints were targeted for cleaning on quartz glass using a peelable coating. First, silicone grease was dissolved in toluene, while SiO_2_ particles (15.0 nm in diameter) were dispersed in anhydrous ethanol. These dispersions were then used to contaminate clean quartz glass, along with fingerprints, to create contaminated quartz glass. Next, a polymer solution was applied to the contaminated glass, allowed to dry completely, and then peeled off to remove the contaminants. Finally, cleaning comparison photographs were obtained using an optical microscope (EVDM10103-M32, Easy View, Shenzhen, Guangdong, China), and the transmission spectra of the quartz glass before and after cleaning were obtained using a UV-Vis-NIR spectrophotometer (LAMBDA750S, PerkinElmer, Waltham, MA, USA).

## 3. Results and Discussion

**Figure 2:**
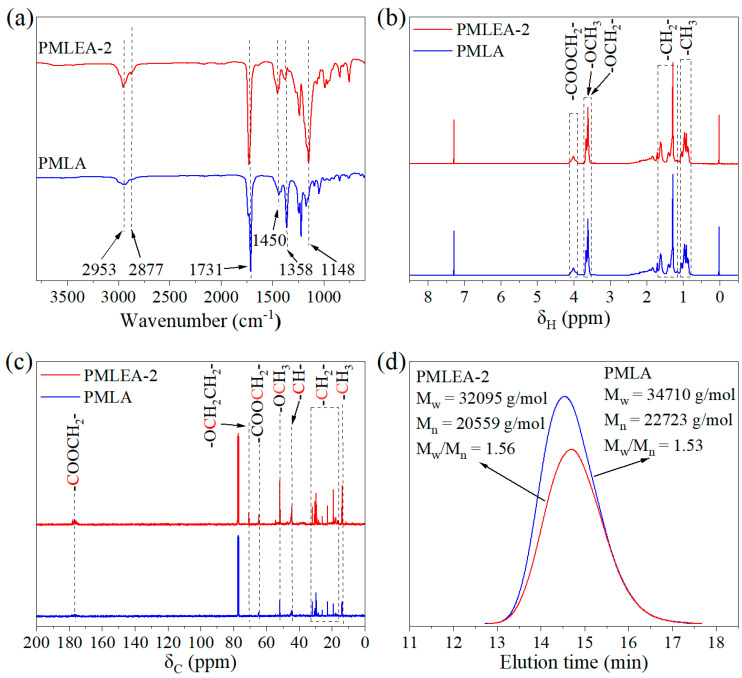
(**a**) FTIR spectra, (**b**) ^1^H-NMR spectra, (**c**) ^13^C-NMR spectra, and (**d**) GPC chromatograms of polyacrylates PMLA and PMLEA-2.

Figure 2a displays the FTIR spectra of the polyacrylate samples PMLA and PMLEA-2. The peaks observed at 2953 and 1358 cm^−1^ correspond to the stretching and deformation vibrations of methyl (-CH_3_) groups, respectively. The stretching vibration and deformation vibration of the methylene (-CH_2_) group are present at 2877 and 1450 cm^−1^, respectively. The characteristic peaks of the stretching vibration of carbonyl (C=O) and ether (C-O-C) groups are observed at 1731 and 1148 cm^−1^, respectively. The pronounced enhancement of the peak at 1148 cm^−1^ in sample PMLEA-2 is attributed to the presence of polyethylene oxide groups in ER-10.

Figure 2b,c depict the ^1^H-NMR and ^13^C-NMR spectra of the polyacrylate samples PMLA and PMLEA-2, respectively. The peaks at δ_H_ = 3.61 ppm and δ_C_ = 51.73 ppm are the corresponding hydrogen and carbon shift peaks of the -OCH_3_ groups in the polyacrylates, and the peaks at δ_H_ = 4.01 ppm and δ_C_ = 64.50 ppm are the chemical shifts of hydrogen and carbon of the -CH_2_- adjacent to the lipid group in the polyacrylates. The peak at δ_C_ = 176.73 ppm is the carbon shift peak of the lipid group -COO- in the copolymer. Additionally, the ^13^C-NMR spectrum of PMLEA-2 exhibits a chemical shift of 70.48 ppm for vinyl oxide. Meanwhile, the proportion of each copolymer component was estimated from the characteristic peak areas in the ^1^H NMR spectra (Figure S3) of the polyacrylate samples. The results are shown in Table S1. It can be seen that the ER-10 content in the polyacrylates is directly proportional to the formulated amount. Notably, the percentage of ER-10 in the synthesized polyacrylates is significantly lower than in the mixture of the monomers. This is due to ER-10’s significantly lower reactivity in comparison to other monomers.

As depicted in Figure 2d, which shows the GPC chromatograms of the polyacrylate samples PMLA and PMLEA-2 (please see more details in Figure S4). The number average molecular weight of the polyacrylate samples exceeds 2.0 × 10^5^ g/mol. Furthermore, the occurrence of chain development during the copolymerization of the four monomers in a one-pot synthesis resulted in a diverse distribution of polymer chains. Despite this, a molecular weight distribution below 1.6 and a single peak-shaped molecular weight distribution were observed in each polyacrylate sample, which indicates that these four monomers have good copolymerization ability, suggesting that the polyacrylates should exhibit the desired tensile and viscoelastic properties for use as a coating.

**Figure 3:**
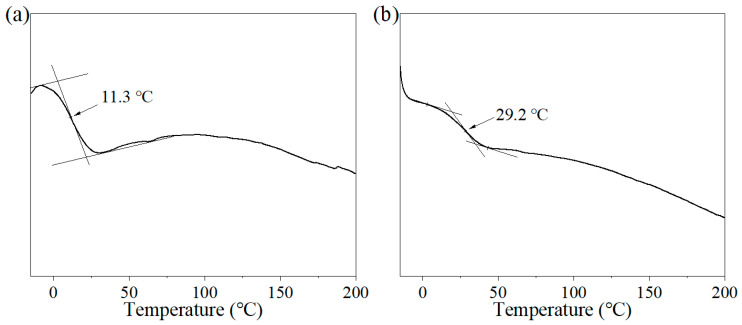
DSC thermograms of polyacrylates: (**a**) PMLA and (**b**) PMLEA-2.

Figure 3 presents the DSC thermograms of the polyacrylate samples PLMA and PMLEA-2, which show that the glass transition temperatures of PMLA and PMLEA-2 are 11.3 and 29.2 °C, respectively. The increase in the glass transition temperature indicates the increase in the rigidity of the polymer chain segments and the consequent increase in the strength of the polyacrylate coating. This is due to the high molecular weight of ER-10 monomers. As a result, the flexibility of the polymer chain of PMLEA-2 is reduced. In addition, the hydroxyl groups in ER-10 readily form hydrogen bonds, which greatly increase the rigidity of the polymer chains. The glass transition temperature of the sample PLMEA-2 is higher than room temperature, which is ideal for the coating’s practical use.

**Figure 4:**
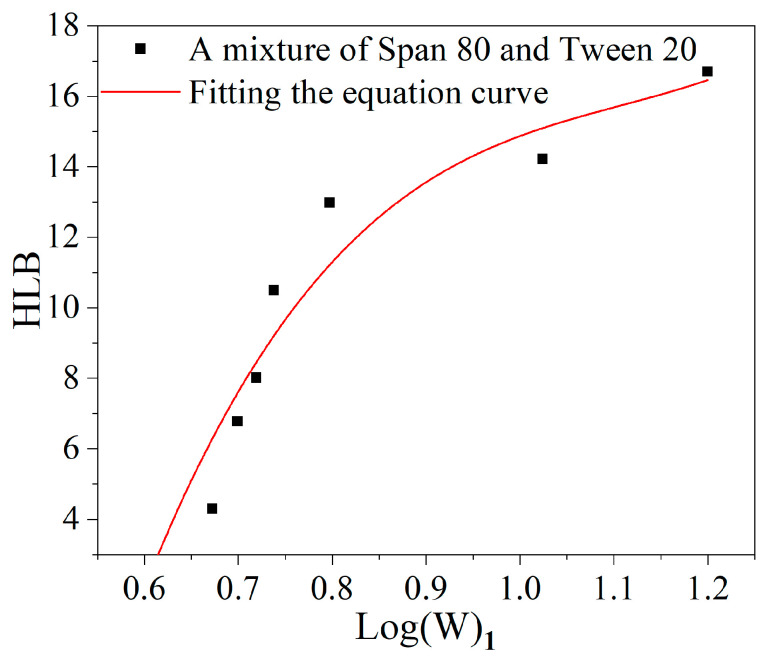
Log(W_1_) versus HLB of Span 80 and Tween 20 in a mixed solution of 1,4-dioxane/toluene.

The relationship between the log(W_1_) and HLB of Span 80 and Tween 20 in a mixed solution of 1,4-dioxane/toluene is depicted in Figure 4, with the red curve representing the fitted equation. The curve equation is as follows:

(2) $$Y=76.52x^{3}-254.36x^{2}+289.00x-95.228$$

The corresponding HLB values are obtained by substituting the water volume value in each polyacrylate sample in Equation (2) and are listed in Table 2. The ratio of hydrophilic to lipophilic groups in the polymer determines the magnitude of the HLB values, and a larger HLB value denotes a more lipophobic and hydrophilic polymer. Therefore, to design an amphiphilic polymer with the effect of reducing surface tension, the HLB value of the polymer should be maintained within a reasonable range. In the sample PMLA, there is no presence of hydrophilic groups, resulting in an HLB value of 0 [41]. Moreover, the hydrophilicity of the polyacrylates increases as the percentage of ER-10 in the samples increases, while simultaneously, the lipophilicity of the polyacrylate samples decreases. An HLB value between 7 and 9 indicates that the polyacrylate sample possesses excellent infiltration capacity, facilitating the effective penetration and wetting of stains for optimal cleaning results [43].

**Figure 5:**
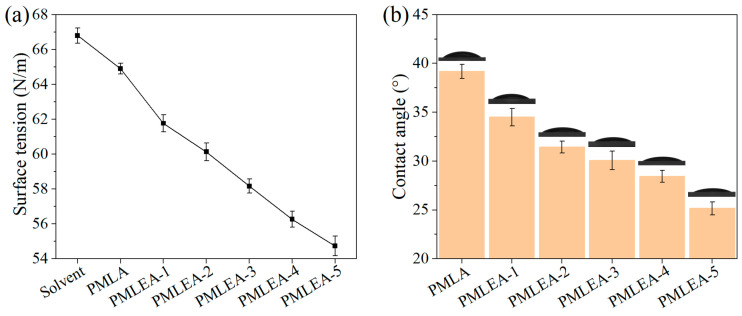
(**a**) Surface tension values of the polymer solutions; (**b**) contact angles of the polymer solution on quartz glasses.

Figure 5a presents the surface tension results of the polymer solutions. The addition of a polyacrylate resulted in a decrease in the solution’s surface tension. Moreover, an increased concentration of ER-10 in the polyacrylate contributed to a reduction in the surface tension of the resultant polymer solution, enhancing its wettability. Consequently, the polymer solution spreads more easily on the substrate surface. Figure 5b presents the contact angles of the polymer solutions on the surface of quartz glass. A larger contact angle indicates the lower surface wettability of a polymer solution. This reduces the contact area between the contaminant and the coating, leading to less effective contaminant removal. However, the contact angles of the six polymer solutions on the quartz glass surface are all less than 40°, indicating that all of them can effectively wet the surface of a clean quartz glass. A polyacrylate with higher hydrophilicity possesses better wettability on a clean quartz glass surface, making it easier to spread on a quartz glass surface. This is consistent with the results of the surface tension of the polymer solutions.

**Figure 6:**
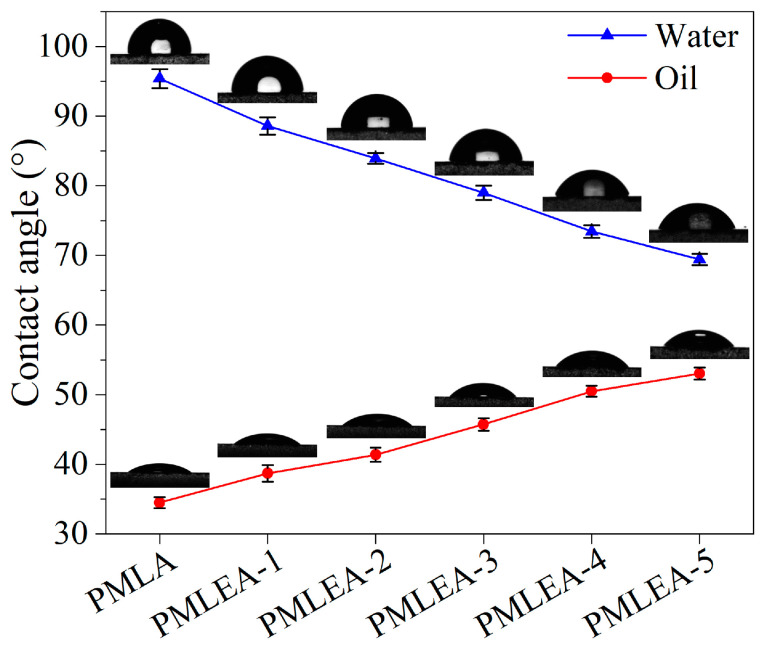
Contact angles of the polyacrylate coatings.

The lower the contact angle between water or oil on its surface, the more hydrophilic or lipophilic the coating is. This promotes easier wetting of stains, allowing contaminants to adhere more effectively to the coating or be partially encapsulated by the coating. In the contact angle test depicted in Figure 6, due to the predominance of lipophilic monomers in the resultant polyacrylates, the coatings exhibit significant lipophilicity. They can effectively impregnate and coat oily stains. However, as the amount of ER-10 increases and the amount of LA decreases, the hydrophilicity of the polyacrylate samples increases while their lipophilicity decreases. This indicates a reduction in the polyacrylate’s ability to adhere to oily stains, while its ability for aqueous stains increases. Furthermore, the water contact angles of the coatings are greater than 65°, providing temporary protection to the substrate from secondary contamination by airborne moisture.

Table 3 provides the mechanical characterization results of the polyacrylate coatings. The PMLA coating is softer, with a tensile strength of just 1.35 MPa and an elongation at break of 275%. In comparison, the PLMEA-5 coating is brittle, with a tensile strength of 3.47 MPa and an elongation at break of 38.3%. This means that substantial variations in the toughness and strength of the coatings were observed in relation to the amount of LA and ER-10 in the polyacrylate samples. LA contributes to coating flexibility, with coating toughness increasing as the amount of LA in the polyacrylate sample rises. However, in the PMLEA series of samples, the flexibility of the polymer molecular chains is diminished due to the high molecular weight of ER-10 monomers. Additionally, the hydroxyl groups in ER-10 readily form hydrogen bonds, significantly enhancing the rigidity of the polymer’s molecular chains. Consequently, coating toughness decreases and strength increases as the ER-10 content in the polyacrylate samples increases. Overall, the polyacrylate samples PMLEA-2 and PMLEA-3 exhibit balanced strength and toughness.

The PMLA coating’s peel strength during coating peeling is just 1.28 N m^−1^. However, an increase in the hydroxyl content of the polyacrylate samples results in the enhanced adhesion of the coatings to the quartz glass surface, leading to an increase in peel strength. As a result, the coating samples PMLEA-5 and PMLEA-4, which exhibited brittleness, tended to break during 180° peeling and could not be completely removed.

**Figure 7:**
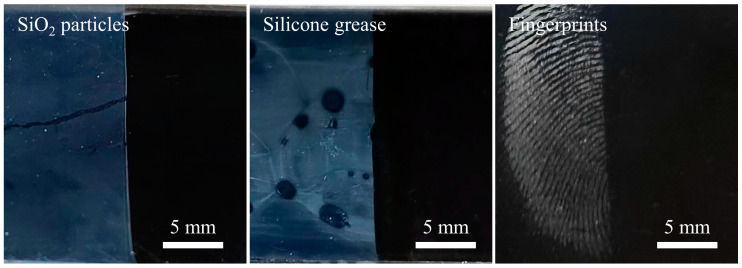
Representative digital photos of quartz glass before and after the removal of surface contamination by PMLEA-2.

Figure 7 presents digital photographs showcasing the representative cleaning effect of PMLEA-2 on three contaminants on quartz glasses. From a macroscopic perspective, the contaminants on the surface of the quartz glass are effectively removed, with clearly defined boundaries between the uncleaned area on the left and the cleaned area on the right. Microphotographs of the boundary areas were utilized to compare the cleanliness of the different samples, as described below.

**Figure 8:**
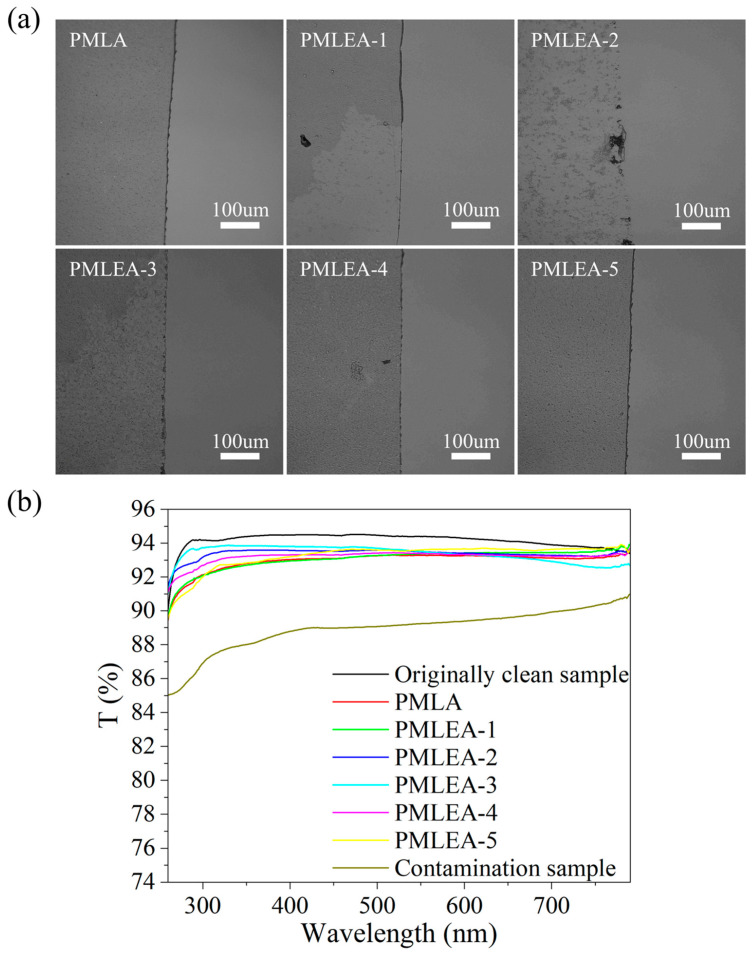
Comparison of the cleaning effect for removing the SiO_2_ particles from the quartz glass: (**a**) the micrographs of the contrast areas and (**b**) the transmission spectra of the quartz glass before and after cleaning.

Figure 8a displays the micrographs of the different polyacrylate samples for cleaning SiO_2_ particles, demonstrating that each sample could effectively remove SiO_2_ particles. In Figure 8b, the UV-Vis-NIR transmission spectra of the quartz glass before and after the cleaning are presented. The transmittance of the quartz glass is reduced after the contamination. However, the transmittance of the cleaned quartz glass can be restored to a level close to that of the initial clean quartz glass. This restoration in transmittance indicates the efficacy of the cleaning process. The low surface tension and good wetting properties of the polyacrylate solutions facilitate the adhesion and encapsulation of the SiO_2_ particles, resulting in a robust cleaning performance.

**Figure 9:**
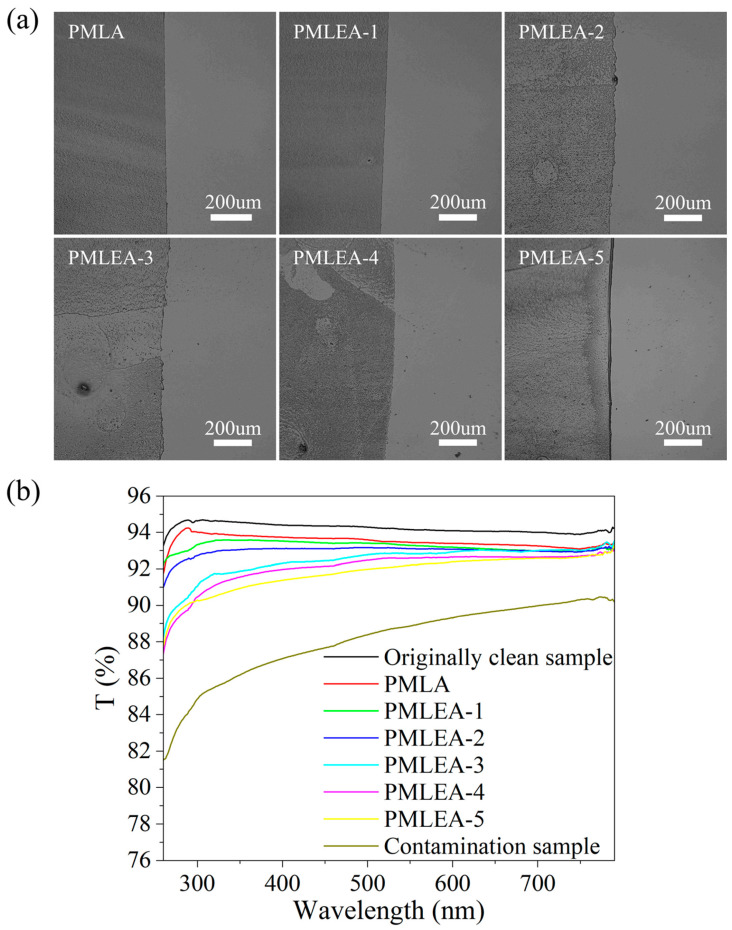
Comparison of the cleaning effect for removing silicone grease from the quartz glass: (**a**) micrographs of the contrast areas and (**b**) transmission spectra of the quartz glass before and after cleaning.

Figure 9a presents the micrographs illustrating the effect of silicone grease removal by the different polyacrylate samples, while Figure 9b displays the UV-Vis-NIR transmission spectra of the quartz glass before and after cleaning with the different polyacrylate samples. As the lipophilicity of the polyacrylate samples decreases, more contaminants remain on the surface of the quartz glass, resulting in decreased cleaning effectiveness. This phenomenon stems from the reduced lipophilicity of the polyacrylate samples, leading to a diminished ability of the polymer solutions to wet and penetrate oily contamination. Consequently, some of the polymer solutions are unable to fully cover and penetrate the surface of the contaminants and the contaminants nestled within, thereby compromising the cleaning efficacy of some of the polyacrylate samples.

**Figure 10:**
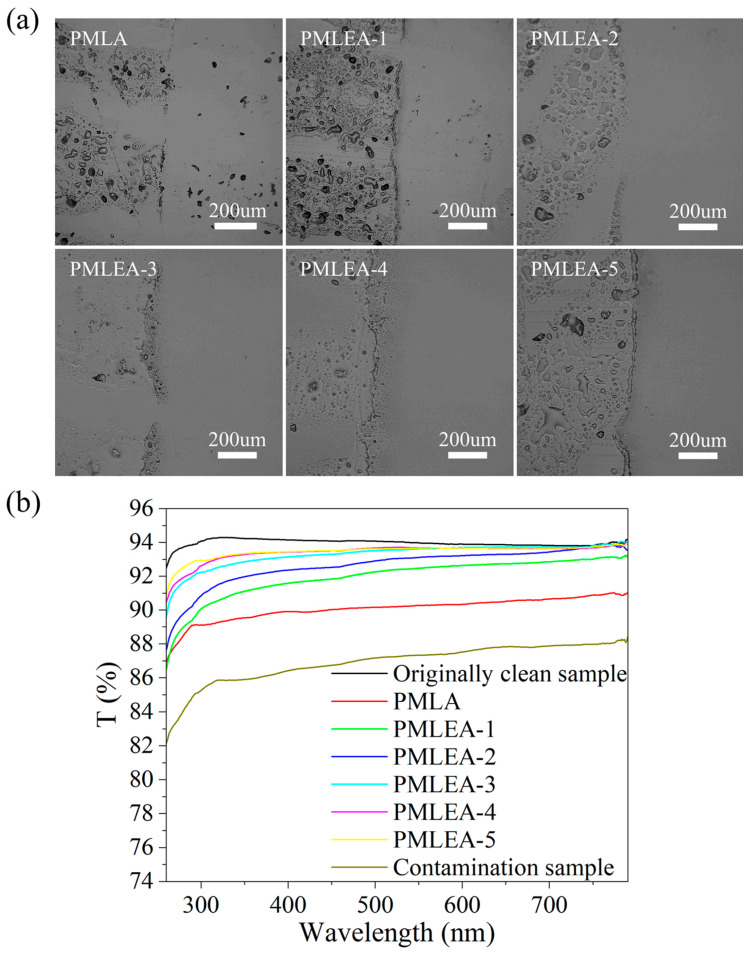
Comparison of the cleaning effect for removing fingerprints from the quartz glass. (**a**) the micrographs of the contrast areas and (**b**) the transmission spectra of the quartz glass before and after cleaning.

Figure 10a presents the micrographs illustrating the effect of the polyacrylate samples on the removal of fingerprints, while Figure 10b displays the transmission spectra of the quartz glass before and after cleaning with the different polyacrylate samples. The cleaning effect of fingerprints improves with the increased hydrophilicity of the polyacrylate samples. This improvement can be attributed to the composition of fingerprints, which primarily consist of water, salt, and grease. The hydrophilicity of the polyacrylate samples plays a crucial role in the penetration of water and salt contamination. Additionally, as the polyacrylate samples possess some degree of lipophilicity, they can effectively wet oils and fats present in the fingerprints. Consequently, polyacrylate samples such as PMLA and PMLEA-1, which exhibit low hydrophilicity, are expected to perform poorly in the removal of fingerprints.

## 4. Conclusions

In this study, polyacrylates possessing both hydrophilic and lipophilic properties were synthesized via free radical polymerization. The structural elucidation and determination of molecular weight were conducted through FTIR, NMR, and GPC characterizations. The investigation into the polyacrylate samples’ HLB, surface tension, and wettability allowed for an assessment of the impact of the LA to ER-10 ratio on the hydrophilicity, lipophilicity, and decontamination efficiency of the synthesized polyacrylates. Furthermore, the coating properties of the polyacrylate samples were found to be influenced by the proportions of LA and ER-10. Through comparative testing, it was revealed that the polyacrylate sample PMLEA-2 exhibited effectiveness in cleaning three common types of surface contaminants typically found on optical instruments. Additionally, the films derived from PMLEA-2 demonstrated balanced mechanical properties and ease of peeling, thereby effectively protecting the surface of the optics. Meanwhile, our results suggest that due to the diverse nature of different contaminants, it is recommended to use a combination of two different coatings to achieve the optimal cleaning effect. Most commercial peelable cleaning coatings use nitrocellulose as the film-forming material. In contrast, polyacrylate-based coatings offer enhanced stability and safety. Meanwhile, the Red First Contact Polymer product from Photonic Cleaning Technologies, which uses an inert polymer as the film-forming material, demonstrates superior cleaning performance and solvent formulation. Our coating exhibited overall decent performance but fell short in both of these aspects, highlighting the need for further refinement and improvement.

## Figures and Tables

**Table 1:** Formulations to synthesize acrylate polymers.

Sample	LA (g)	ER-10 (g)	MMA (g)	BA (g)
PMLA	7.00	0	12.00	8.00
PMLEA-1	3.50	3.50		
PMLEA-2	2.34	4.66		
PMLEA-3	1.75	5.25		
PMLEA-4	1.40	5.60		
PMLEA-5	1.17	5.83		

**Table 2:** Volumes of water in the polymer samples and the corresponding HLB values.

Sample	W_2_ (mL)	Log(W_2_)	HLB
PMLA	3.6	0.56	0
PMLEA-1	4.2	0.62	4.61
PMLEA-2	4.8	0.68	7.78
PMLEA-3	5.2	0.71	9.39
PMLEA-4	5.4	0.73	10.1
PMLEA-5	5.8	0.76	11.2

**Table 3:** Mechanical properties of polyacrylate coatings.

Sample	Stress (MPa)	Strain (%)	Peel Strength (N m^−1^)
PMLA	1.35 ± 0.07	275 ± 9	1.28 ± 0.11
PMLEA-1	1.95 ± 0.12	258 ± 7	1.78 ± 0.08
PMLEA-2	2.57 ± 0.04	183 ± 5	2.07 ± 0.11
PMLEA-3	2.87 ± 0.11	110 ± 2	2.46 ± 0.08
PMLEA-4	3.23 ± 0.07	76.6 ± 4.1	2.94 ± 0.13
PMLEA-5	3.47 ± 0.09	38.3 ± 3.7	3.20 ± 0.09

## Data Availability

All the data generated or analyzed during this study are included in this published article and its supplementary information files.

## Supplementary Materials

The following supporting information can be downloaded at: https://www.mdpi.com/article/10.3390/ma17194813/s1, Figure S1: Preparation process of polyacrylates. Figure S2: (a) FTIR spectrum of ER-10, (b) ^1^H-NMR spectrum of ER-10. Figure S3: ^1^H-NMR spectra of the copolymer samples. Figure S4: GPC chromatograms of the copolymers. Table S1: Areas of the characteristic peaks.

## References

[1] Ling X.L., Zhao Y.N., Li D.W., Shao J.D., Fan Z.X. (2009). Damage investigations of AR coating under atmospheric and vacuum conditions. Opt. Laser Technol..

[2] Li X.G., Shen J. (2011). The stability of sol–gel silica coatings in vacuum with organic contaminants. J. Sol-Gel Sci. Technol..

[3] Cui Y., Zhao Y.N., Yu H., He H.B., Shao J.D. (2008). Impact of organic contamination on laser-induced damage threshold of high reflectance coatings in vacuum. Appl. Surf. Sci..

[4] Yang L., Xiang X., Miao X.X., Li Z.J., Li L., Yuan X.D., Zhou G.R., Lv H.B., Zu X.T. (2015). Influence of oil contamination on the optical performance and laser induced damage of fused silica. Opt. Laser Technol..

[5] Bude J., Carr C.W., Miller P.E., Parham T., Whitman P., Monticelli M., Raman R., Cross D., Welday B., Ravizza F. (2017). Particle damage sources for fused silica optics and their mitigation on high energy laser systems. Opt. Express.

[6] Ristau D., Jupé M., Starke K. (2009). Laser damage thresholds of optical coatings. Thin Solid Films.

[7] Lielienfeld P. (1986). Optical Detection of Particle Contamination of Surface: A Review. Aerosol Sci. Technol..

[8] Ye Y.Y., Yuan X.D., Xiang X., Cheng X.F., Miao X.X. (2012). Laser cleaning of particle and grease contaminations on the surface of optics. Optik.

[9] Bennett J.M. (2003). How to clean surfaces. Laser-Induced Damage in Optical Materials, Proceedings of the 2003 (XXXV Annual Symposium on Optical Materials for High Power Lasers: Boulder Damage Symposium), Boulder, CO, USA, 22–24 September 2003.

[10] Bennett J.M. (2005). Dos and don’ts in characterizing and cleaning optical surfaces. Optical Fabrication, Testing, and Metrology II, Proceedings of the Optical Systems Design 2005, Jena, Germany, 12–16 September 2005.

[11] Houston K.D. (2006). Comparative mirror cleaning study: A study on removing particulate contamination. Optical Systems Degradation, Contamination, and Stray Light: Effects, Measurements, and Control II, Proceedings of the SPIE Optics + Photonics, San Diego, CA, USA, 13–17 August 2006.

[12] Kimura W.D., Kim G.H., Balick B. (1994). Comparison of laser and CO_2_ snow cleaning of astronomical mirror samples. Advanced Technology Optical Telescopes V, Proceedings of the 1994 Symposium on Astronomical Telescopes and Instrumentation for the 21st Century), Kailua-Kona, HI, USA, 13–18 March 1994.

[13] Máša V., Horňák D., Petrilák D. (2021). Industrial use of dry ice blasting in surface cleaning. J. Clean. Prod..

[14] Lobmeyer L., Carey L. (2018). Optical cleaning to remove particles for JWST mirror surfaces. Systems Contamination: Prediction, Control, and Performance 2018, Proceedings of the SPIE Optical Engineering + Applications, San Diego, CA, USA, 19–23 August 2018.

[15] Rigolet F. (1999). Cleaning and surface preparation: Which lasers for which applications. Assem. Autom..

[16] Mann K., Wolff-Rottke B., Mu F. (1996). Cleaning of optical surfaces by excimer laser radiation. Appl. Surf. Sci..

[17] Lizon J.L., Deiries S. (2014). Cleaning of extremely sensitive optical surfaces. Advances in Optical and Mechanical Technologies for Telescopes and Instrumentation, Proceedings of the SPIE Astronomical Telescopes + Instrumentation, Montréal, QC, Canada, 22–27 June 2014.

[18] Gossen K., Ehrmann A. (2019). Influence of FTO glass cleaning on DSSC performance. Optik.

[19] Barthel A.J., Luo J.W., Hwang K.S., Lee J.-Y., Kim S.H. (2016). Boundary lubrication effect of organic residue left on surface after evaporation of organic cleaning solvent. Wear.

[20] Dey T., Naughton D. (2016). Cleaning and anti-reflective (AR) hydrophobic coating of glass surface: A review from materials science perspective. J. Sol-Gel Sci. Technol..

[21] Wagle P.G., Tamboli S.S., More A.P. (2021). Peelable coatings: A review. Prog. Org. Coat..

[22] Joseph R. (2004). The Lowdown on Peelable Coatings Specs and Usage. Met. Finish..

[23] Gardon J.L. (1963). Peel adhesion. II. A theoretical analysis. J. Appl. Polym. Sci..

[24] Liprandi D., Bosia F., Pugno N.M. (2020). A theoretical-numerical model for the peeling of elastic membranes. J. Mech. Phys. Solids.

[25] Cipriano J. (2005). Cleaning Modern Antireflection-Coated Optics with Collodion. Cloudy Nights Telescope Reviews (Blog). https://www.cloudynights.com/articles/cat/articles/how-to/collodion-optics-cleaning-r1265.

[26] Baker M.I., Walsh S.P., Schwartz Z., Boyan B.D. (2012). A review of polyvinyl alcohol and its uses in cartilage and orthopedic applications. J. Biomed. Mater. Res. Part B Appl. Biomater..

[27] Rao S.V.S., Lal K.B. (2004). Surface decontamination studies using polyvinyl acetate based strippable polymer. J. Radioanal. Nucl. Chem..

[28] Lewandowski K., Krepski L.R., Mickus D.E. (2004). Dry-peelable temporary protective coatings from waterborne self-crosslinkable sulfourethane–silanol dispersions. J. Appl. Polym. Sci..

[29] Long N.H., Park H.W., Chae G.S., Lee J.H., Bae S.W., Shin S. (2019). Preparation of peelable coating films with a metal organic framework (UiO-66) and self-crosslinkable polyurethane for the decomposition of methyl paraoxon. Polymers.

[30] He Z.Y., Li Y.T., Xiao Z.Q., Jiang H., Zhou Y.L., Luo D.L. (2020). Synthesis and Preparation of (Acrylic Copolymer) Ternary System Peelable Sealing Decontamination Material. Polymers.

[31] Wang X.R., Ma G.Y., Ma H.F., Wu Z. (2021). Stripping and mechanical properties of water-based polyacrylate stripper and its application in art masking fluid. Iran. Polym. J..

[32] Xu X.R., Li J., Pan X.H., Lin X.Y., Li Z.G. (2023). Preparation of high tensile strength modified cellulose-based strippable coating and its application on radioactive decontamination at low-temperature. Mater. Today Commun..

[33] Tang J.L., Li S.Y., Wang Y.Y., Zhang H.L., Lin B., Sun M.X. (2022). Ethyl cellulose based peelable coatings with visual sensing of hydrogen sulfide. Prog. Org. Coat..

[34] Kohli R. (2019). Applications of Strippable Coatings for Removal of Surface Contaminants. Developments in Surface Contamination and Cleaning: Applications of Cleaning Techniques.

[35] Von Rybinski W. (2007). Physical Aspects of Cleaning Processes. Handbook for Cleaning/Decontamination of Surfaces.

[36] Song Y., Zhou G.Y., Tu M.T., Zhang J.C., Wang P. (2021). Effectiveness of oolong tea and simethicone solution for lens cleansing during colonoscopy: A double-blinded randomized study. Medicine.

[37] Michon B., López-Sánchez U., Degrouard J., Nury H., Leforestier A., Rio E., Salonen A., Zoonens M. (2023). Role of surfactants in electron cryo-microscopy film preparation. Biophys. J..

[38] Anton W.L., Spinelli H.J., Patil A.A. (1998). Acrylic Polymer Compounds. U.S. Patent.

[39] Yamashita K., Matsuki M., Asai H., Matsuyama N., Tojo H., Kurota H., Akasaka K., Obara H. (2002). Aqueous Dispersion of a Peelable Coating Composition. U.S. Patent.

[40] Pasquali R.C., Sacco N., Bregni C. (2009). The studies on hydrophilic-lipophilic balance (HLB): Sixty years after William C. Griffin’s pioneer work (1949–2009). Lat. Am. J. Pharm..

[41] Griffin W.C. (1954). Calculation of HLB values of non-ionic surfactants. J. Cosmet. Sci..

[42] Greenwald H.L., Brown G.L., Fineman M.N. (1956). Determination of hydrophile-lipophile character of surface active agents and oils by water titration. Anal. Chem..

[43] Ananthapadmanabhan K.P. (1993). Surfactant Solutions: Adsorption and Aggregation Properties. Interactions of Surfactants with Polymers and Proteins.

