# Supplementary material for: Synthesis of Amphiphilic Polyacrylates as Peelable Coatings for Optical Surface Cleaning
Source: Materials (Basel). 2024 Sep 30;17(19):4813. doi: 10.3390/ma17194813 (PMC11477594; doi:10.3390/ma17194813)
Supplement: Supplementary file 1 [file materials-17-04813-s001.zip › materials-3206044-supplementary.pdf]

Supporting Information

1. Preparation Process of Polyacrylates.

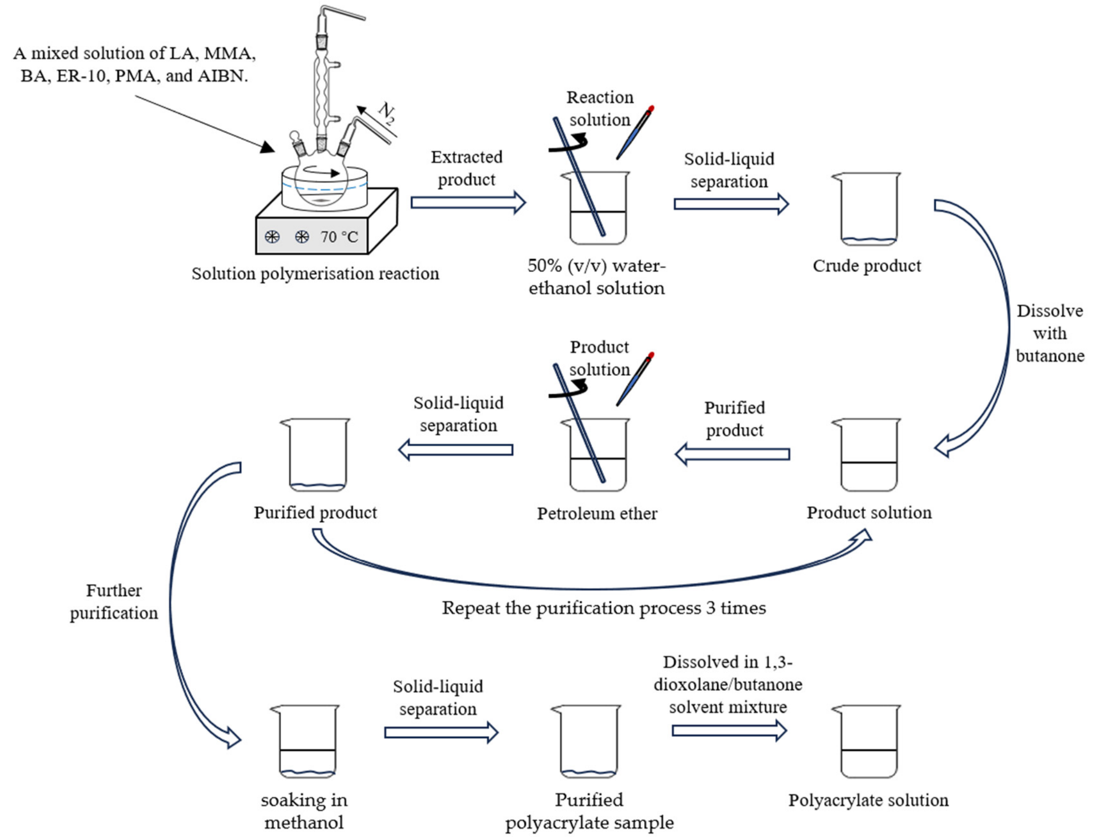

Figure S1. Preparation process of polyacrylates.

2. Characterization of ER-10

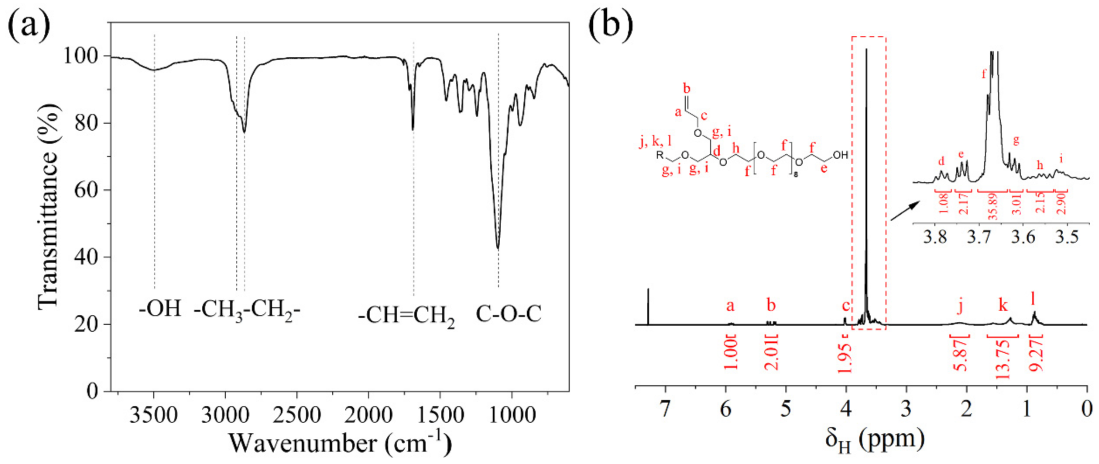

Figure S2. (a) FTIR spectrum of ER-10, (b) ^{1}H-NMR spectrum of ER-10.

3. ^{1}H-NMR of Copolymer Samples

Figure S3 shows the ^{1}H-NMR spectra of the copolymer samples. The

characteristic peak at 1.09 ppm corresponds to $-CH_3$, the characteristic peaks appearing at 3.61 ppm belong to $-OCH_3$ and $-OCH_2-$, and the chemical shift of $-CH_2-$, which is adjacent to the lipid group, is at 4.01 ppm. The result allows for the evaluation of the copolymer composition by the area of the characteristic peaks. The area of $-CH_3$ at 1.09 ppm is used in a 1:1 relationship with the area of $-OCH_3$ at 3.61 ppm. The relative composition of ER-10 in the polyacrylates can be derived by subtracting the area of $-OCH_3$ from the area of the b-peak. The formula is given in Equation (S1). Similarly, the ratio of the sum of LA and BA to MMA in the polyacrylate constituents can be estimated from the ratio of the area of the a-peak to the area of the c-peak. The results are shown in Table S1.

$$\frac{S_{(b)} - S_{(C)}}{47} = \frac{n_{(MMA)}}{n_{(ER-10)}} \qquad (S1)$$

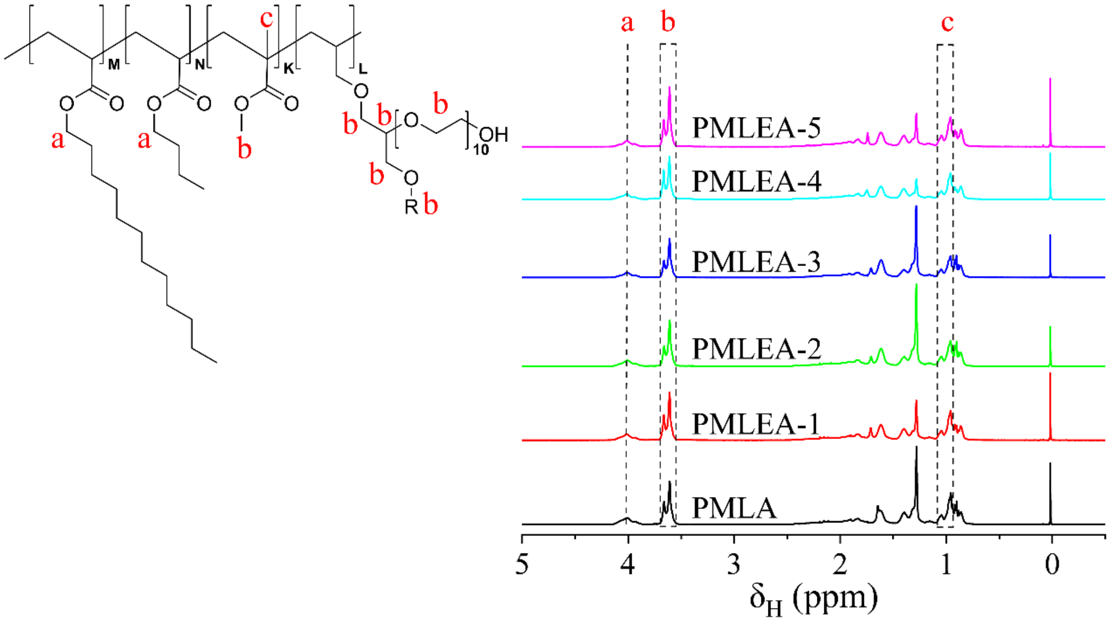

Figure S3. ^{1}H-NMR spectra of the copolymer samples.

Table S1. Areas of the characteristic peaks.

Sample	a	b	c	Ingredient ratio (%) LA and BA: MMA: ER-10	Molar ratio of monomer addition (%) LA and BA: MMA: ER-10
PMLA	1.32	3.03		40: 60: 0	43.26: 56.74: 0
PMLEA-1	1.24	3.32		38.11: 61.46: 0.43	38.23: 59.58: 2.19
PMLEA-2	1.17	3.53	3.00	36.65: 62.66: 0.69	36.47: 60.57: 2.96
PMLEA-3	1.13	3.67		35.78: 63.33: 0.89	35.53: 61.10: 3.37
PMLEA-4	1.09	3.76		34.91:64.06:1.03	34.96: 61.43: 3.61
PMLEA-5	1.05	3.87		34.02:64.81:1.17	34.57: 61.65: 3.78

4. GPC Characterization of the Copolymers.

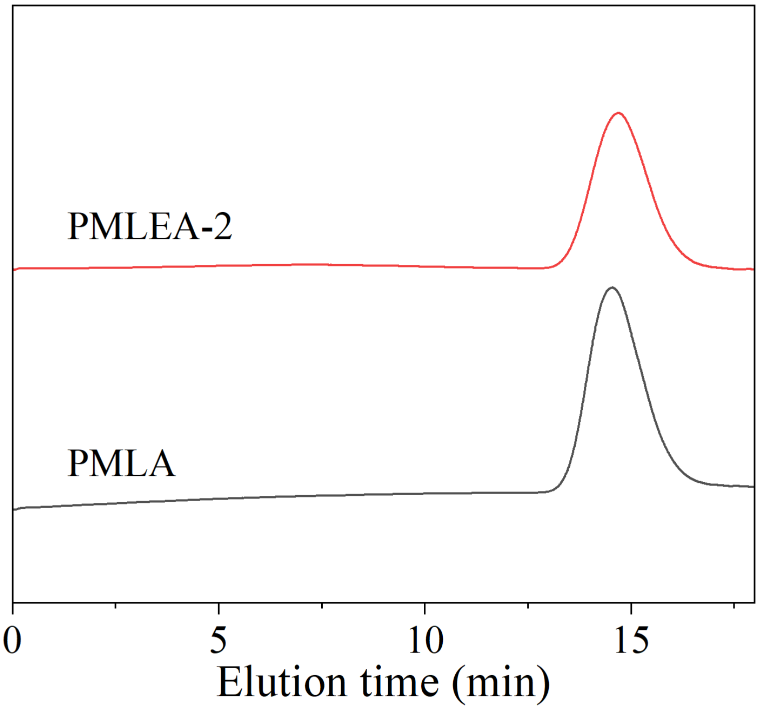

Figure S4. GPC chromatograms of the copolymers.
